# 40 Years of the APOC Partnership

**DOI:** 10.1371/journal.pntd.0003562

**Published:** 2015-05-14

**Authors:** Jean-Baptist Roungou, Laurent Yameogo, Chris Mwikisa, Daniel A. Boakye, Donald A. P. Bundy

**Affiliations:** 1 World Health Organization/African Programme for Onchocerciasis Control (WHO/APOC), Ouagadougou, Burkina Faso; 2 World Bank, Washington, D.C., United States of America; Lindsley F. Kimball Research Institute, New York Blood Center, UNITED STATES

The fight against onchocerciasis (river blindness), one of the most devastating neglected tropical diseases (NTDs), has mobilized significant resources and brought together diverse public and private stakeholders. Affected communities, governments of endemic countries, non-governmental development organizations (NGDOs), donors, and researchers are contributing, each in their own way, to what is considered today as one of the major public health achievements of recent decades in Africa [1]. Onchocerciasis is losing ground, and its elimination in Africa is now possible within a reasonable timeframe [2,3].

Onchocerciasis is a vector-borne disease caused by the filarial worm *Onchocerca volvulus*. The Onchocerciasis Control Programme in West Africa (OCP), launched in 1974, initially focused its activities on vector control in 11 West African countries and invested US$556 million over 28 years [4]. OCP succeeded in controlling river blindness in ten countries, with an amazing 20% economic rate of return [5]. In 1987, in light of demonstrated ivermectin efficacy and safety in humans, Merck & Co. Inc. committed to providing the medicine free of charge to endemic countries for as long as necessary to eliminate river blindness as a public health problem. This historic pledge enabled a new era in river blindness control through mass drug administration [6].

Building upon OCP, the African Programme for Onchocerciasis Control (APOC) was launched in 1995 to extend the gains in river blindness control achieved in West Africa to the 19 remaining endemic countries, mainly located in central and eastern Africa. APOC adopted the community-directed treatment with ivermectin (CDTi) approach as its core strategy. The effectiveness of CDTi in improving coverage and compliance has since been demonstrated, and it is now used as a model for scaling up other public health interventions [7].

APOC’s work is underpinned by four main pillars: (i) ivermectin donation by Merck; (ii) commitment to the CDTi strategy; (iii) a unique partnership among the affected communities, governments, donors, NGDOs, and APOC Secretariat; and (iv) continued scientific (basic and operational) research, results of which were immediately ploughed in to improve performance. Structures and mechanisms have been set up to implement, manage, and review this work.

APOC’s work translates on the ground into CDTi projects, which were 107 in 2012. The CDTi projects deliver ivermectin once a year to the affected and at-risk communities. These communities are fully empowered to play their role: they select community-directed ivermectin distributors (CDDs), decide on the most suitable period for ivermectin mass distribution, monitor ivermectin distribution and provide incentives to CDDs, and report treatment adverse effects to the nearest health facilities. On their side, the health workers who are trained by experts from APOC and in-country consultants ensure training of CDDs and their supervision; they help with management of ivermectin supply and take charge of responding to severe adverse effects of ivermectin treatment. This way, CDTi enormously contributes to primary health care implementation.

The control of river blindness has been a remarkable success story. Now the focus has shifted to a more ambitious goal: the elimination of river blindness in addition to other preventable NTDs from Africa. To help endemic countries achieve this aim, plans are underway to transition APOC into a new regional NTD entity, provisionally named Programme for the Elimination of Neglected Tropical Diseases (PENDA), in 2016. As river-blindness–endemic countries in Africa engage in the elimination of the disease and APOC transforms into a new regional entity, it is important to widely share the APOC partnership experience. This series of articles will highlight the different aspects of the fight, focusing on the history and governance of APOC, the management of the ivermectin donation [8], the technical foundations of the work of APOC, the country programs and perspectives, NGDOs action [9], mechanisms for financial sustainability [10], and the future of regional NTD elimination efforts.
